# Therapeutic potential of young plasma in reversing age-related liver inflammation via modulation of NLRP3 inflammasome and necroptosis

**DOI:** 10.1007/s10522-025-10260-9

**Published:** 2025-05-26

**Authors:** Burcu Baba, Taha Ceylani, Hikmet Taner Teker, Seda Keskin, Aysun Inan Genc, Rafig Gurbanov, Eda Acikgoz

**Affiliations:** 1 Department of Medical Biochemistry, Faculty of Medicine, Yuksek Ihtisas University, Ankara, Turkey; 2 Department of Molecular Biology and Genetics, Faculty of Science and Literature, Mus Alparslan University, Mus, Turkey; 3 Department of Food Quality Control and Analysis, Mus Alparslan University, Mus, Turkey; 4 Department of Medical Biology and Genetics, Faculty of Medicine, Ankara Medipol University, Ankara, Turkey; 5 Department of Histology and Embryology, Faculty of Medicine, Van Yuzuncu Yil University, 65090 Van, Turkey; 6 Department of Biology, Faculty of Natural Sciences, Kastamonu University, Kastamonu, Turkey; 7 Department of Bioengineering, Bilecik Seyh Edebali University, Bilecik, Turkey; 8 Central Research Laboratory, Bilecik Seyh Edebali University, Bilecik, Turkey; 9 Department of Neuroscience, Faculty of Medicine, Van Yuzuncu Yil University, Van, Turkey

**Keywords:** Inflammaging, Plasma exchange, Infrared spectroscopy, Necroptosis, NLRP3 Inflammasome

## Abstract

The phenomenon of inflammaging, characterized by an increase in low-grade chronic inflammation, is closely associated with diseases related to liver dysfunction. This study investigated daily plasma exchange between 5-week-old and 24-month-old Sprague Dawley rats for 30 days, focusing on protein secondary structures, NLRP3 inflammasome, and necroptosis. Conformation changes in protein secondary structures were identified by infrared spectroscopy-based pattern recognition analysis. Liver biopsies with histochemical and immunohistochemical staining were used to assess molecules associated with inflammation, necroptosis and NLRP3 inflammasome complex. Expression levels of NLRP3 components were determined by qPCR. Enhanced random coils, 3_10_ helices, β-turns, and loop structures were identified in old rats and young rats with old plasma. Young rats and old rats with young plasma displayed higher α-helices and β-sheet structures. Young rats with old plasma showed increased NLRP3, ASC, caspase-1, IL-1β, and IL-18 mRNA levels, indicating an inflammatory response. Whereas old rats with young plasma exhibited lower inflammation levels. Histological evaluations revealed that young rats receiving aged plasma showed significantly increased levels of NLRP3, ASC, caspase-1, IL-1β, TNF-α, VEGFR2, RIPK1, and MLKL immunoreactivity, whereas decreased immunoreactivity in aged rats receiving young plasma. These findings suggest that young plasma reduces NLRP3 inflammasome activation and necroptosis in aged rats.

**Supplementary Information:**

The online version contains supplementary material available at 10.1007/s10522-025-10260-9.

## Introduction

“Inflammaging,” which refers to the chronic low-grade inflammation observed during the aging process, is strongly associated with age-related diseases (Sabira Mohammed et al. 2021a, b). Damaged cells release pro-inflammatory cytokines like interleukin-6 (IL-6), tumor necrosis factor-alpha (TNF-α), and interleukin-1 beta (IL-1β), contributing to the chronic inflammatory process associated with aging (Gao et al. 2023) The NLRP3 inflammasome is a key innate immune complex that detects cellular stress and promotes maturation of pro-inflammatory cytokines like IL-1β and IL-18. Activation of the NLRP3 inflammasome not only drives pro-inflammatory cytokine release but also intersects with necroptotic signaling pathways, creating a feed-forward loop that exacerbates liver injury. This mechanistic crosstalk highlights the importance of targeting both inflammasome activation and necroptosis to effectively mitigate hepatic inflammaging (Almalki & Almujri 2025; Li et al. 2023).

Recent research has identified necroptosis as a form of cell death that causes inflammation by releasing damage-associated molecular patterns (DAMPs). During aging, DAMPs released from damaged hepatocytes are significant factors in the development of chronic inflammation in the aged liver (Thadathil et al. 2021). In addition, damaged hepatocytes and fibroblasts in the aging liver may release age-related cytokines induced by DAMPs during the aging process, resulting in changes in collagen density. As a result, hepatic fibrosis indicates the development of inflammation in the aging liver (Lin et al. 2024). Necroptosis can be induced by specific interactions between membrane receptors and ligands and is also driven by inflammation, aging, and autoimmune diseases (Sabira Mohammed et al. 2021a, b). This cell death type is mediated by a necrosome comprising receptor-interacting serine/threonine-protein kinases 1 and 3 (RIPK1, RIPK3) and mixed lineage kinase domain-like protein (MLKL) (Salvadores & Court 2020). Particularly during aging, DAMPs are significant activators of the NLRP3 inflammasome, as they bind to pattern recognition receptors (PRRs) like toll-like receptors (TLRs) on innate immune cells. IL-1β, which matures due to binding, causes inconsistent activation of NLRP3 inflammasome complexes and exacerbates chronic inflammation in aging (Wree et al. 2014).

Given the central role of inflammasome activation and necroptosis in age-related liver pathology, interventions capable of modulating these pathways are of significant therapeutic interest. Young plasma therapy, which has shown systemic rejuvenating effects, presents a promising approach to attenuate these molecular drivers of hepatic dysfunction. Besides, therapeutic strategies to increase longevity and slow aging have become popular (Ceylani et al. 2023; Huang et al. 2023). One of these approaches that has recently attracted attention is the effects of young blood plasma transfer on limiting or reversing aging in various organs in preclinical studies (Ceylani et al. 2023; Hosseini et al. 2023). Studies investigating the effects of systemic and immunogenic components in young blood plasma on aging and longevity have positively affected the lifespan of older animals (Ceylani et al. 2023; Kheifets & Braithwaite 2019; B. C. Lee et al. 2019). The underlying mechanisms are because young plasma contains numerous rejuvenating factors that promote tissue regeneration or reduce pathological degeneration (Kheifets & Braithwaite 2019; A. D. Liu et al. 2018). However, one of the molecular mechanistic aspects of young plasma that has not yet been elucidated is how young plasma interacts with the NLRP3 inflammasome and necroptosis signaling, a cell death pathway, in the aged liver. In the context of aging-related liver dysfunction, structural integrity of proteins is often compromised due to chronic inflammation and stress-induced cell death processes like necroptosis (Diaz-Villanueva et al. 2015; Sabira Mohammed et al. 2021a, b). These biological events are closely associated with alterations in protein secondary structures, such as a shift from well-ordered α-helices and β-sheets to disordered elements like random coils and β-turns, reflecting impaired proteostasis (Lindner & Demarez 2009; Taylor & Dillin 2011). Fourier-transform infrared (FTIR) spectroscopy is a valuable tool for analyzing protein secondary structure, particularly its sensitivity to vibrational modes of the peptide backbone in the amide I and II regions, providing unique spectral signatures for different secondary structures, including alpha-helices, beta-sheets and unordered structures (Dong et al. 1990; S. N. Yang et al. 2022).

Pretreatment techniques like second derivative and deconvolution, coupled with pattern recognition approaches, enhance the resolution of overlapping bands within the amide I region. This enables the quantification of various secondary structures present in a protein sample (Bozkurt et al. 2012; Dousseau & Pezolet 1990; D. C. Lee et al. 1990; Pribic 1994). These methods are especially useful when X-ray crystallography is not feasible, offering a complementary approach for determining protein structure in their native, aqueous environments (Byler & Susi 1986; Haris & Severcan 1999). Some studies have shown that FTIR spectroscopy, applying second derivative and Fourier self-deconvolution techniques, can distinguish subtle differences in protein secondary structures (Bozkurt et al. 2012; Gurbanov et al. 2016; S. N. Yang et al. 2022). This spectroscopic approach allows for detailed analysis of secondary structural dynamics underlying cellular stress responses. The changes in structural motifs observed via FTIR spectroscopy can be linked to necroptotic activity and inflammaging, as the functional roles of proteins like RIPK1, RIPK3, and MLKL depend on their secondary structure integrity (Berghe et al. 2014; Martinez-Osorio et al. 2023). We consider this property to be fundamental in studying processes such as aging, necroptosis, and inflammation, where changes in protein conformation can drive disease mechanisms (Holehouse & Kragelund 2024; Rinauro et al. 2024).

Building upon our previous findings that demonstrated the ability of young plasma to reverse chronic hepatic inflammation in aged rats (Teker et al. 2023), the current study seeks to provide a deeper mechanistic understanding of how young plasma influences two pivotal molecular pathways implicated in age-related liver dysfunction: The NLRP3 inflammasome axis and the necroptosis signaling pathway. Both the NLRP3 inflammasome and necroptosis have been increasingly recognized as key contributors to hepatic inflammaging, fibrosis, and progressive organ failure in elderly populations. However, the potential of young plasma to modulate these specific pathways, thereby attenuating inflammatory and necrotic cell death processes in the aging liver, has not been thoroughly investigated to date. To address this gap, our study employs an integrated approach combining infrared (IR) spectroscopy-based pattern recognition, which allows non-invasive molecular fingerprinting of tissue alterations, with targeted immunohistochemical and molecular analyses to quantify the expression and localization of critical components of the NLRP3 inflammasome complex and necroptosis markers. Through this multifaceted methodological framework, we aim to delineate the specific interactions between young plasma therapy and the molecular cascades driving inflammation and necroptosis in the senescent liver, thereby offering new insights into potential plasma-based therapeutic interventions for age-related hepatic dysfunction.

## Material & methods

### Experimental design

This study used male Sprague Dawley (SD) rats as model organisms. Pooled plasma (0.5 ml per day for 30 days, intravenously (i.v.) into the tail vein) obtained from young (5 weeks, n = 51) rats was administered to the aged rats (n = 6; 24 months). Similarly, pooled plasma (0.25 ml per day for a month, i.v. into the tail vein) obtained from old rats (24 months, n = 16) was administered to young rats (5 weeks, n = 6). All animals were housed with free access to food and water in standard animal care facilities, under a 12-h light/dark cycle at a constant 21 °C.

This study was approved by the Saki Yenilli Experimental Animal Production and Practice Laboratory Ethics Committee (approval number: 2021/03), and all experimental protocols were carried out in accordance with institutional guidelines and the ARRIVE (Animal Research: Reporting of In Vivo Experiments) guidelines (https://arriveguidelines.org). The volume of blood plasma transferred was determined as 1/10th of the animal’s total blood plasma volume (Villeda et al. 2014). The young and old rats used in the study were obtained from a certified center for the production and study of experimental animals. Rats in each group were housed in separate Plexiglas cages (6 rats per cage) to ensure no cross-contamination, and all co-housed rats remained in the same experimental group throughout the study. No signs of allergic reactions or plasma rejection were observed during the exchange process, and no animals were lost during the experiment. After the treatment period, all experimental and control animals were lightly anesthetized with ether and subsequently sacrificed. Liver tissues were collected immediately, shocked on dry ice, and stored at -80 °C until further analysis.

### Plasma collection and administration

Pooled rat plasma was collected by terminal cardiac puncture at the time of euthanasia. Plasma was prepared from blood collected with EDTA followed by centrifugation at 1000 g. For plasma denaturation, plasma was heated for 2–3 min at 95 °C, followed by a short spin at 1000 g. All plasma aliquots were stored at − 80 °C until use. Before administration, plasma was dialyzed using 3.5 kDa D-tube dialyzers (EMD Millipore) in PBS to remove EDTA (Villeda et al. 2014). The selection of plasma volumes was guided by previous plasma exchange studies in rodent models (Pei et al. 2024; Villeda et al. 2014), aiming to achieve effective systemic exposure without exceeding safe transfusion limits. For aged rats, 0.5 ml of pooled young plasma per day was administered intravenously for 30 days, corresponding to approximately 10% of the rat’s total plasma volume, a dose shown to induce measurable biological effects while avoiding circulatory overload. In the reciprocal experiment, 0.25 ml per day was administered to young rats, scaled proportionally to their lower body weight and blood volume. This protocol was designed to balance efficacy with safety and maintain consistency with established methodologies in plasma transfer research (Ceylani & Teker 2022).

### Infrared spectral measurements and pattern recognition analysis of protein secondary structures

Liver samples were examined using an ATR-FTIR spectrometer (PerkinElmer) according to the previously reported methodology as described in Teker et al. (Teker et al. 2023). Principal Component Analysis (PCA) is a pattern recognition approach that can be used for protein secondary structure determination (Haris & Severcan 1999). PCA was conducted on liver infrared (IR) spectra after different pretreatment steps using the Unscrambler® X 10.3 (CAMO Software AS, Norway) software. First, the raw IR data were subjected to baseline offset transformation in the 4000–650 cm⁻^1^ spectral window. Subsequently, the second data derivatives were obtained using the Savitzky-Golay transformation method in the 1700–1600 cm⁻^1^ spectral window of Amide I protein. Finally, the second derivative spectra were transformed using the unit vector normalization method. The line plot of the pre-treated dataset used in PCA analysis is given in Fig S1. PCA was run on a mean-centered second derivative data matrix in the amide I band’s 1700–1600 cm⁻^1^ IR window to obtain information on protein secondary structures. The singular value decomposition (SVD) algorithm was applied to a full cross-validated dataset. Hotelling’s T-squared distribution test was used within the 99.9% confidence range (shown as an ellipse in the scores plot). The PCA results are presented as scores and loadings plots (Izgordu et al. 2024; Şenol et al. 2021).

### Masson’s trichrome (MT) staining

MT staining method was used to demonstrate the deposition density of collagen fibers in rat liver. The other liver tissue biopsies were embedded in paraffin blocks and cut at a thickness of 5 µm and used for MT staining (Bio-Optica, Cat No: 04–010802, Milan, Italy) according to the instructions in the kit. All staining procedures were performed at room temperature. For quantification of MT staining intensity, a similar grayscale binary separation technique with the lowest threshold level was used to identify blue-stained areas, and signal intensities from five images of each group were analyzed with ImageJ (Fiji). This quantification method was adapted from Adomshick et al. (Adomshick et al. 2020). Microphotographs were obtained from a Nikon Eclipse Ni microscope (Tokyo, Japan) equipped with a camera (Nikon DS-Fi2, Japan) and imaging software (NIS Elements F 4.00.00, Nikon Soft Imaging Solution, Japan) at 400X magnification.

### Immunohistochemical (IHC) staining

IHC was performed to elucidate the protective effects of young plasma on necroptosis markers (MLKL, RIPK1), NLRP3 inflammasome signaling pathway (NLRP3, ASC, IL-1β), the inflammatory cytokine marker (TNF-α), and the vascularization marker (VEGFR2), which previously described the protocol for paraffin sections (Ceylani et al. 2023). After deparaffinization, endogenous peroxidase activity was inhibited by incubation with 3% hydrogen peroxide for 10 min and samples were washed with PBS. Heat-induced antigen retrieval was performed by incubation in citrate buffer, and the sections were incubated in a blocking solution for 10 min to prevent non-specific binding. Following the blocking step, all sections were incubated at + 4 °C overnight with anti-MLKL (Elabscience, E-AB-67102, dilution rate: 1:200), anti- RIPK1 (Elabscience, E-AB-18284, dilution rate: 1:200), anti-NLRP3 (Elabscience, E-AB-93112, dilution rate: 1:200), anti-ASC (Elabscience, E-AB-30582, dilution rate: 1:200), anti-IL-1β (Santa Cruz (11E5), sc-52012, dilution rate:1:200), anti- TNF-α (Bioss, BS-10802R, dilution rate: 1:200), and anti-VEGFR2 (Bioss, BS-10412, dilution rate:1:200) polyclonal antibodies. The next day, biotinylated antibodies (TP-125-BN, Thermo Scientific) and streptavidin peroxidase (TS-125-HR) were applied to the sections. Then, a 3,3′- Diaminobenzidine substrate kit (ab64238, Abcam) was performed for 3–5 min. Finally, the sections were counterstained with Mayer’s haematoxylin and visualized under a light microscope (Olympus BX53, Japan) using a camera (Olympus DP27, Japan) and microscope imaging systems (Olympus cellSens Entry, Japan).

For immunohistochemical evaluation, in areas selected by random sampling, immunprecipitation of stained cells which were calculated to their immunoreactivity intensities were applied with Image J (Fiji) and were statistically evaluated. The measurements of MLKL, RIPK1, NLRP3, ASC, IL-1β, TNF-α, and VEGFR2 expression intensities (% Area) in the liver sections were performed over 10 random areas of interest (3 different sections for each rat per group) (Ceylani et al. 2023).

### RNA isolation and cDNA synthesis

The extraction of RNA from tissue samples was carried out using the GeneAll® Hybrid-R™ kit (Cat. No. 305–101, Korea). The NanoDrop QC SkanIt software 4.1 was utilized on a 96-well plate spectrophotometer (Multiskan GO, Thermo Scientific, Waltham, MA, USA) to evaluate the integrity and concentration of the extracted RNA. After RNA isolation, the synthesis of cDNA was performed using the ProtoScript First Strand cDNA Synthesis Kit (SuScript cDNA Synthesis Kit, Catalog No: RT01A025, Türkiye), following the manufacturer’s protocol. This process involved an initial denaturation step at 70 °C for 5 min, a 1-h incubation period at 42 °C, and enzyme inactivation at 80 °C for 5 min. The Sensoquest Thermocycler Labcycler with Thermoblock 96 Gold Plated Silver 012–103 (Germany) facilitated this thermal cycling process. The synthesized cDNA was then stored at − 20 °C for future genetic analyses, ensuring its integrity and suitability for additional studies.

### Primers and gene expression analysis

The primers utilized in this study were designed using the Primer3 software (http://primer3.ut.ee/). Quantitative real-time PCR (qPCR) analysis was conducted employing the SuScript 1-Step SYBR qPCR Kit (Catalog No: RT01A046, 200Rxn, Ankara, Turkey) on a Rotor-Gene Q system (QIAGEN, Hilden, Germany), with 0.1 mL strip tubes and a final reaction volume of 10 µL. The thermal cycling conditions were as follows: 50 °C for 2 min, 95 °C for 1 min, followed by 40 cycles of 95 °C for 10 s and 60 °C for 30 s. GAPDH served as the internal reference gene for normalization. Although the widely adopted 2^–ΔΔCT^ method (Livak & Schmittgen 2001) was initially referenced, the expression data in this study were analyzed using a modified normalization strategy to facilitate individual-level statistical comparisons. Specifically, ΔCT values were calculated for each sample as CT_target—CT_reference. To normalize inter-group variation and enable statistical testing on individual biological replicates (n = 5 per group), each of which was analyzed in triplicate (technical replicates), each ΔCT value was divided by the mean ΔCT of the corresponding control group. This normalization approach preserves within-group variance and facilitates graphical representation of individual data points, as suggested in (Schmittgen & Livak 2008). This method provides a transparent and statistically traceable alternative to the ΔΔCT approach, particularly in small-sample experiments where averaging across groups may obscure biologically relevant variability. All statistical analyses were conducted using normalized ΔCT values, and results are reported accordingly. Primer sequences are provided in Table 1.

**Table 1:** Sequences of specific primers

Gene name	Elongation Position	Sequence (5′-3′)	Reference
NLRP3	Forward Reverse	TGTGAGAAGCAGGTTCTACTCT GGATGCTCCTTGACCAGTTGG	(Ydens et al. 2015)
ASC	Forward Reverse	CAGCACAGGCAAGCACTCA GGTGGTCTCTGCACGAACT	(Ydens et al. 2015)
Caspase-1	Forward Reverse	GGGACCCTCAAGTTTTGCC GACGTGTACGAGTGGTTGTATT	(Ydens et al. 2015)
IL-1β	Forward Reverse	GCAACTGTTCCTGAACTCAACT ATCTTTTGGGGTCCGTCAACT	(Ydens et al. 2015)
IL-18	Forward Reverse	GACTCTTGCGTCAACTTCAAGG CAGGCTGTCTTTTGTCAACGA	(Ydens et al. 2015)
Glyceraldehyde-3-phosphate dehydrogenase (Gapdh)	Forward Reverse	TGGACCTCATGGCCTACATG AGGGAGATGCTCAGTGTTGG	(Kwon et al. 2021)

Gapdh was used as a reference.

### Hematological analysis

Blood samples were collected under euthanasia and centrifuged at 3500 rpm for 15 min, then serum was separated at 4 °C by a refrigerated centrifuge. Red blood cell (RBC, 10^6^/uL), hematocrit (HCT, %), hemoglobin (HGB, g/dL), mean corpuscular volume (MCV, fL), mean corpuscular hemoglobin (MCH, pg), mean corpuscular hemoglobin concentration (MCHC, g/dL), red cell distribution width (RDW, fL), mean platelet volume (MPV, fL), platelets (PLT, 10^3^/uL), platelet distribution width (PDW, fL) and white blood cell (WBC, 10^3^/uL) were determined using an automated hematology analyzer (Mindray Automated Analyzer) following the manufactural instructions.

### Statistical analysis

Statistical evaluations and graph plots of the results were performed using GraphPad Prism version 10.01 (GraphPad Software, USA). The normality of the data was assessed prior to further analysis. Depending on the distribution and comparison design, either one-way ANOVA followed by Tukey’s post hoc test or unpaired Student’s *t*-test was applied to evaluate differences among the experimental groups: YC (young control), YOP (young with old plasma), OC (old control), and OYP (old with young plasma). Results are expressed as mean ± standard error of the mean (SEM). Statistical significance levels were denoted as follows: *p* ≤ 0.05 *, *p* ≤ 0.01 **, *p* ≤ 0.001 ***, and *p* ≤ 0.0001 ****.

## Results

### Effects of plasma exchange on protein secondary structures in liver

**Fig. 1:**
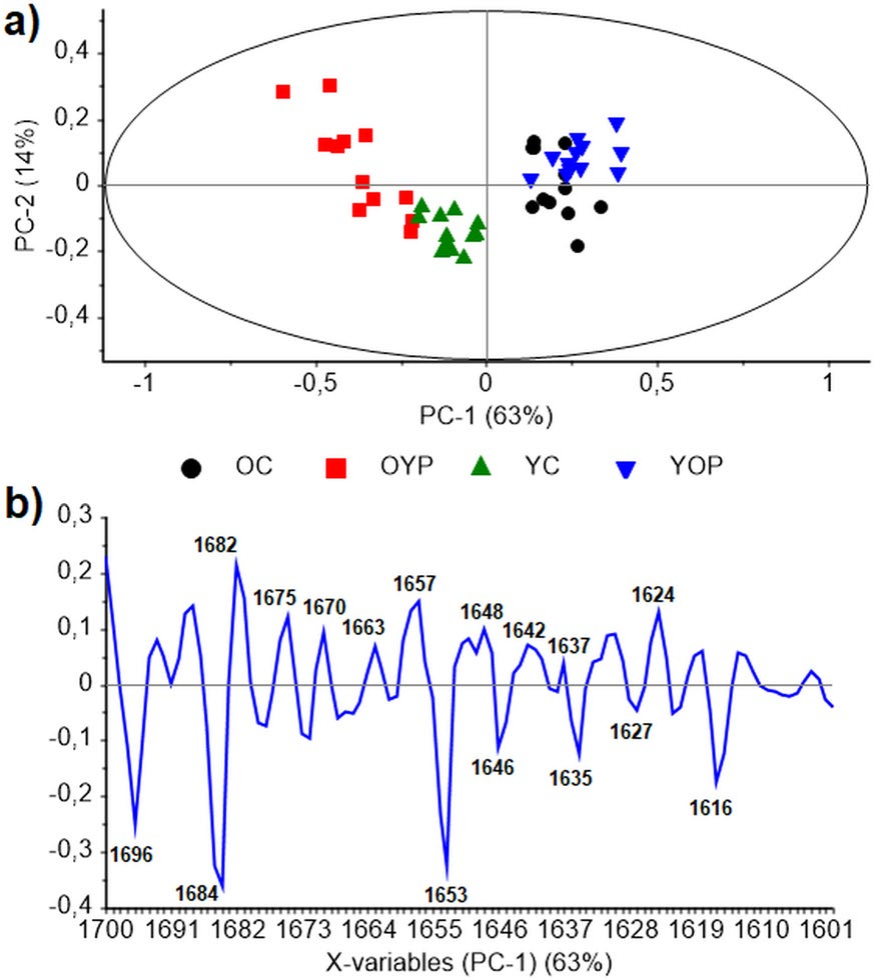
Principal Component Analysis describes how the protein secondary structures undergo conformational changes due to plasma transfer in the liver. **a** The scores plot demonstrates the interaction between experimental group members on the PC-1 and PC-2 coordinates. **b** The loadings plot explains the behavior of spectral discriminators for protein secondary structures. The analysis was conducted in the second-derivative and vector-normalized Amide I (1700–1600 cm.^−1^) spectral window. YC (young control); YOP (young with old plasma); OC (old control); OYP (old with young plasma)

The impact of plasma exchange between old and young rats on protein secondary structures of the liver was determined by the Principal Component Analysis (PCA). This Pattern Recognition Technique utilized pretreated infrared (IR) spectral data of Amide I protein region (1700–1600 cm^−1^) from liver tissues of all animals as a data matrix. The scores plot demonstrates the restoring capacity of young plasma and vice versa for old plasma. Technically, the young rats receiving old plasma (YOP group) clustered together with the members of old rat controls (OC group) along the right positive side of the PC-1 coordinate. Conversely, the OYP (old rats receiving young plasma) group members moved toward the YC (young rat controls) group and completely clustered on the left negative PC-1 coordinate (Fig. 1a).

The protein secondary structures are explained from the positive and negative discriminators revealed from the loadings plot and associated table (Fig. 1b, Table S1). For the interpretation of loadings features; variables that are positive discriminators (those that contribute positively) in the loadings plot are associated with points on the positive side of the scores plot. Similarly, variables that are negative discriminators (those that contribute negatively) in the loadings plot are associated with points on the negative side of the scores plot. In other words, the positive and negative discriminators seen on the plot are positively correlated with the positive and negative sides of the scores plot, respectively (Gurbanov et al. 2021; Şenol et al. 2021).

The main positive discriminators were encountered at 1682 (β-turn), 1675 (β-turn), 1670 (β-turn), 1663 (3_10_ Helix), 1657 (3_10_ Helix), 1648 (random coil), 1642 (random coil), 1637 (loop structures connecting helices), and 1624 (β-sheet) cm^−1^ spectral positions. These discriminators positively correlated with the scores for young rats receiving old plasma (YOP group) and old rat controls (OC group) located on the positive side of the PC-1 axis. This indicates an increase in β-turns, 3_10_ helices, random coils, and loops connecting helices in both old rats and young rats receiving old plasma. On the other hand, there was a positive correlation between the main negative discriminators at 1696 (antiparallel β-sheet), 1684 (intermolecular β-sheet), 1653 (α-helix), 1646 (α-helix), 1635 (β-sheet), 1627 (β-sheet), and 1616 (intermolecular β-sheet) cm^−1^ spectral positions and the scores of old rats receiving young plasma (OYP group) and young rat controls (YC group) positioned on the negative side of the PC-1 axis. This can be attributed to an increase in various β-sheet elements and α-helices in both young rats and old rats receiving young plasma.

### Effects of young and old plasma transfer on aging-induced hepatic fibrosis

**Fig. 2:**
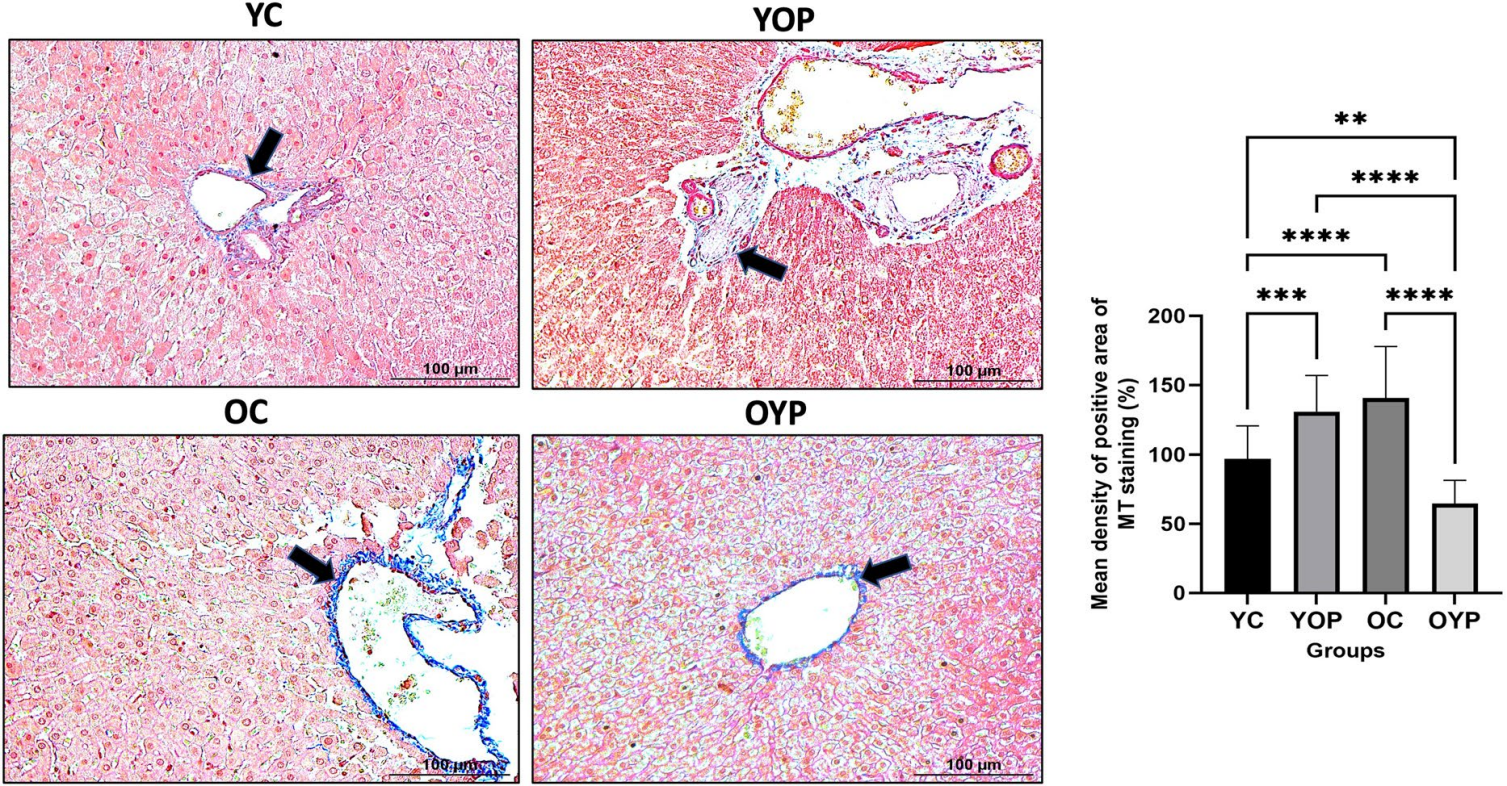
Histological changes associated with liver fibrosis due to plasma transfer in YC, YOP, OC and OYP groups. Representative liver MT-stained liver sections for detection of densities of collagen deposition (using Image J (Fiji)) in areas shown in blue color (black arrows). Magnification, 200 × and 400x. Scale bars, 100 µm and 50 µm. YC (young control); YOP (young with old plasma); OC (old control); OYP (old with young plasma)

We evaluated the effects of plasma transfer on aging-induced hepatic fibrosis, Masson’s trichrome (MS) staining was performed to measure collagen deposition in the liver sections. Our results revealed that collagen deposition was increased in the OC group, but effectively decreased in old rats receiving young plasma (Fig. 2) (****p ≤ 0.0001). Furthermore, the receiving old plasma may promote collagen deposition, leading to fibrosis that increase with aging. This increase in the collagen density in YOP group compared to the YC group showed that it significantly stimulated collagen expression (Fig. 2). These data suggest that young plasma may decrease liver fibrosis from aging (****p ≤ 0.0001).

### Effects of plasma exchange on NLRP3 inflammasome activation in liver tissue

**Fig. 3:**
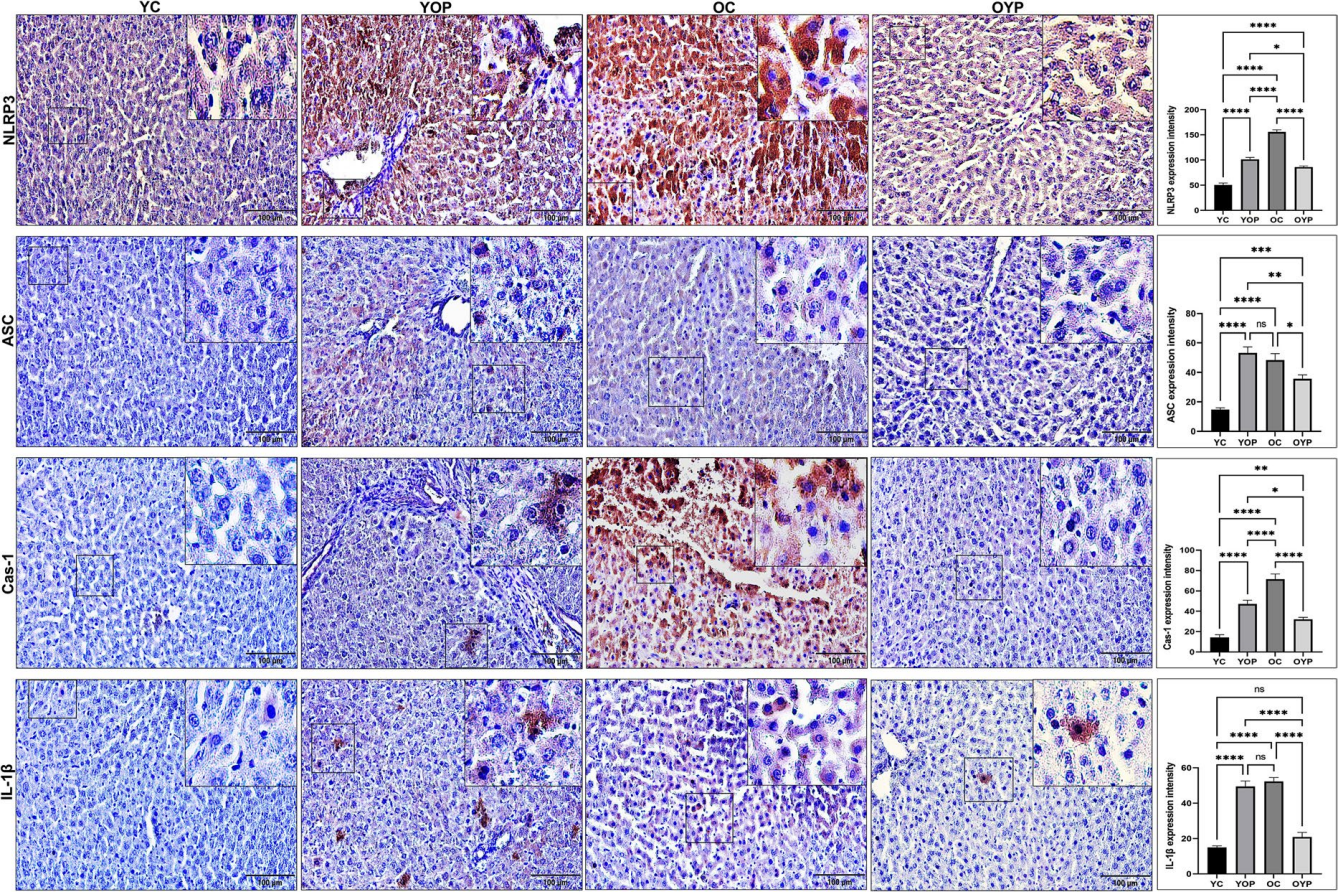
NLP3, ASC, Caspase-1 and IL-1β immunostaining intensities in the liver sections. Zoom images of the relevant areas within each square are given in the upper right corner. Graphs of NLP3, ASC, caspase-1 and IL-1β intensities measured in ImageJ (FIJI) and statistical results are given. Values are expressed as mean ± SEM. The significance levels were stated as *p < 0.05, **p ≤ 0.01 ***p ≤ 0.001 and ****p ≤ 0.0001. Scale bar: 100 μm. NLRP3: NLR family pyrin domain containing 3; ASC: Apoptosis-associated speck-like protein containing a caspase recruitment domain (CARD); IL-1β: Interleukin 1 beta. YC (young control); YOP (young with old plasma); OC (old control); OYP (old with young plasma)

Liver damage from inflammaging and fibrosis requires NLRP3 inflammasome activation. Inflammaging stimulates NLRP3 inflammasome complex (Thadathil et al. 2021; Wree et al. 2014). Therefore, we evaluated the effects of plasma transfer on aging-induced NLRP3 inflammasome activation in the rat liver. In our results, NLRP3 and ASC expressions were significantly increased in the YOP group compared to the YC group, suggesting that the inflammation-causing factors of old plasma induced NLRP3 inflammasome activation (Fig. 3) (****p ≤ 0.0001). However, NLRP3 and ASC expressions were significantly decreased in the OYP group compared to the OC group (****p ≤ 0.0001). Results demonstrated that young plasma’s anti-inflammatory impact reduced NLRP3 inflammasome activation (Fig. 3). Furthermore, Caspase-1 and IL-1β expressions were significantly increased in the YOP group compared to the YC group, whereas Caspase-1 and IL-1β expressions were significantly decreased in the OYP group compared to the OC group (Fig. 3) (****p ≤ 0.0001). Remarkably, NLRP3 and Caspase 1 expression levels appeared to be significantly increased in the OC group compared to the other groups in the region of inflammatory areas (Fig. 3). Taken together, all results indicate that the expression changes in NLP3 inflammasome pathway components triggered inflammaging and were modulated by plasma exchange.

### Young plasma mediates hepatoprotection against age-related necroptosis

**Fig. 4:**
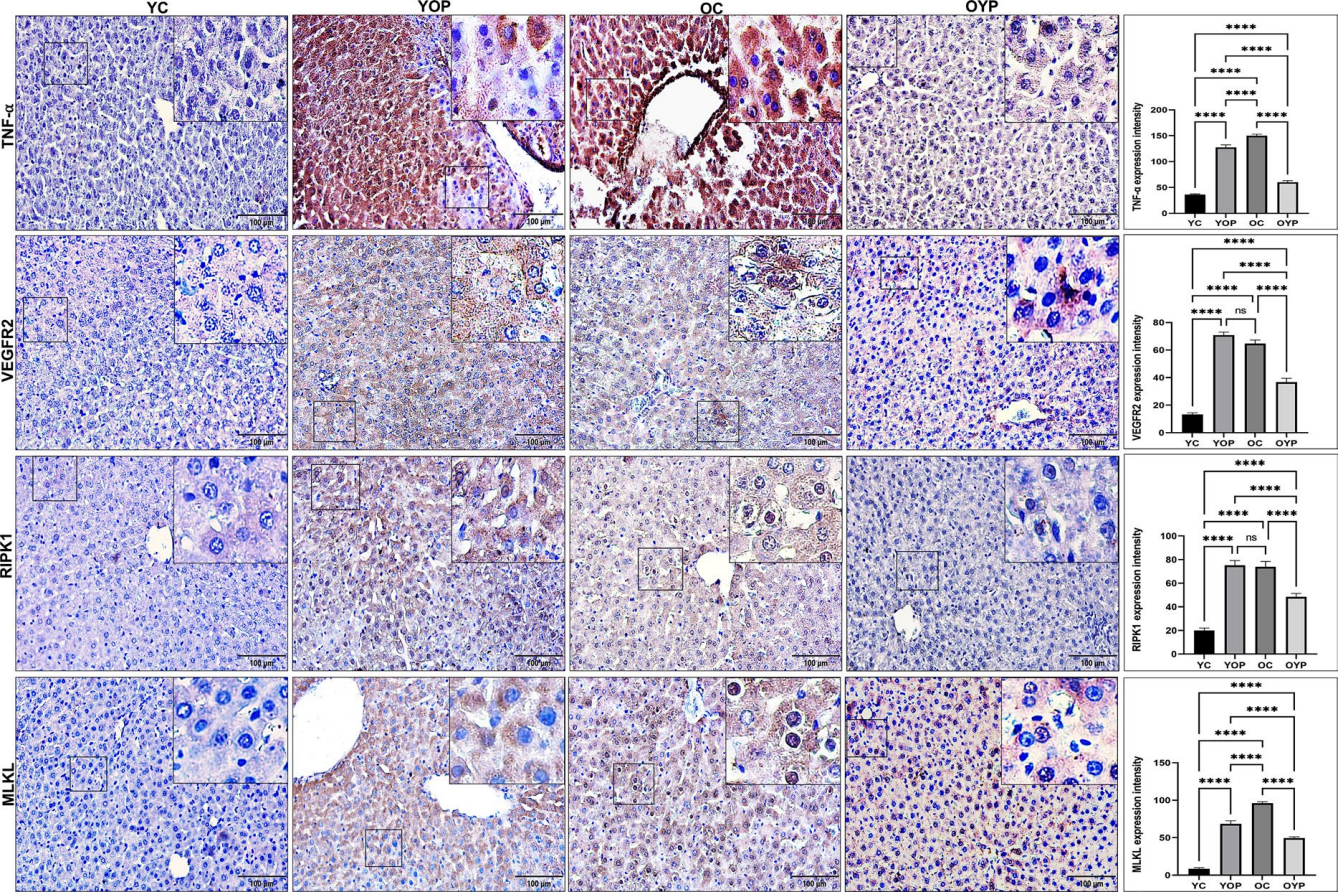
TNF-α, VEGFR2, RİPK1, and MLKL immunostaining intensities in the liver sections. Zoom images of the relevant areas within each square are given in the upper right corner. Graphs of TNF-α, VEGFR2, RİPK1, and MLKL intensities measured in ImageJ (FIJI) and statistical results are given. Values are expressed as mean ± SEM. The significance levels were stated as *p < 0.05, **p ≤ 0.01 ***p ≤ 0.001 and ****p ≤ 0.0001. Scale bar: 100 μm. TNF-α: Tumour Necrosis Factor-alpha, VEGFR2: Vascular Endothelial Growth Factor Receptor Type 2; RİPK1: Receptor-interacting serine/threonine-protein kinase 1; MLKL: Mixed lineage kinase domain like pseudokinase. YC (young control); YOP (young with old plasma); OC (old control); OYP (old with young plasma)

One of the main features of aging is the presence of necroptosis, a regulated form of inflammatory death (Dhuriya & Sharma 2018). In our previous study, histopathological data showed that hepatocytes exhibited necroptotic features due to aging (Teker et al. 2023). Accordingly, in this study, TNF-α, VEGFR2, RIPK1, and MLKL immunoreactivities were evaluated to reveal the effects of plasma exchange on the necroptosis signaling pathway. As seen in Fig. 4, TNF-α, VEGFR2, RIPK1 and MLKL expression levels were significantly increased in the YOP group compared to the YC group (****p ≤ 0,0001). This increase suggests that old plasma triggers the activation of the necroptosis pathway by stimulating TNF-α secretion and promoting VEGFR2 activation as a result of the triggering of inflammaging. Furthermore, TNF-α, VEGFR2, RIPK1, and MLKL expression levels were significantly decreased in the OYP group compared to the OC group (Fig. 4, ****p ≤ 0.0001). It is observed that young plasma suppresses inflammation and improves fibrosis by decreasing the levels of TNF-α, inhibiting VEGFR2 expression and modulating necroptosis signaling. Upon evaluating the acquired results in context, it is understood that plasma transfer regulates the activation of the necroptosis signaling pathway.

### Effects of plasma exchange on NLRP3 inflammasome gene

**Fig. 5:**
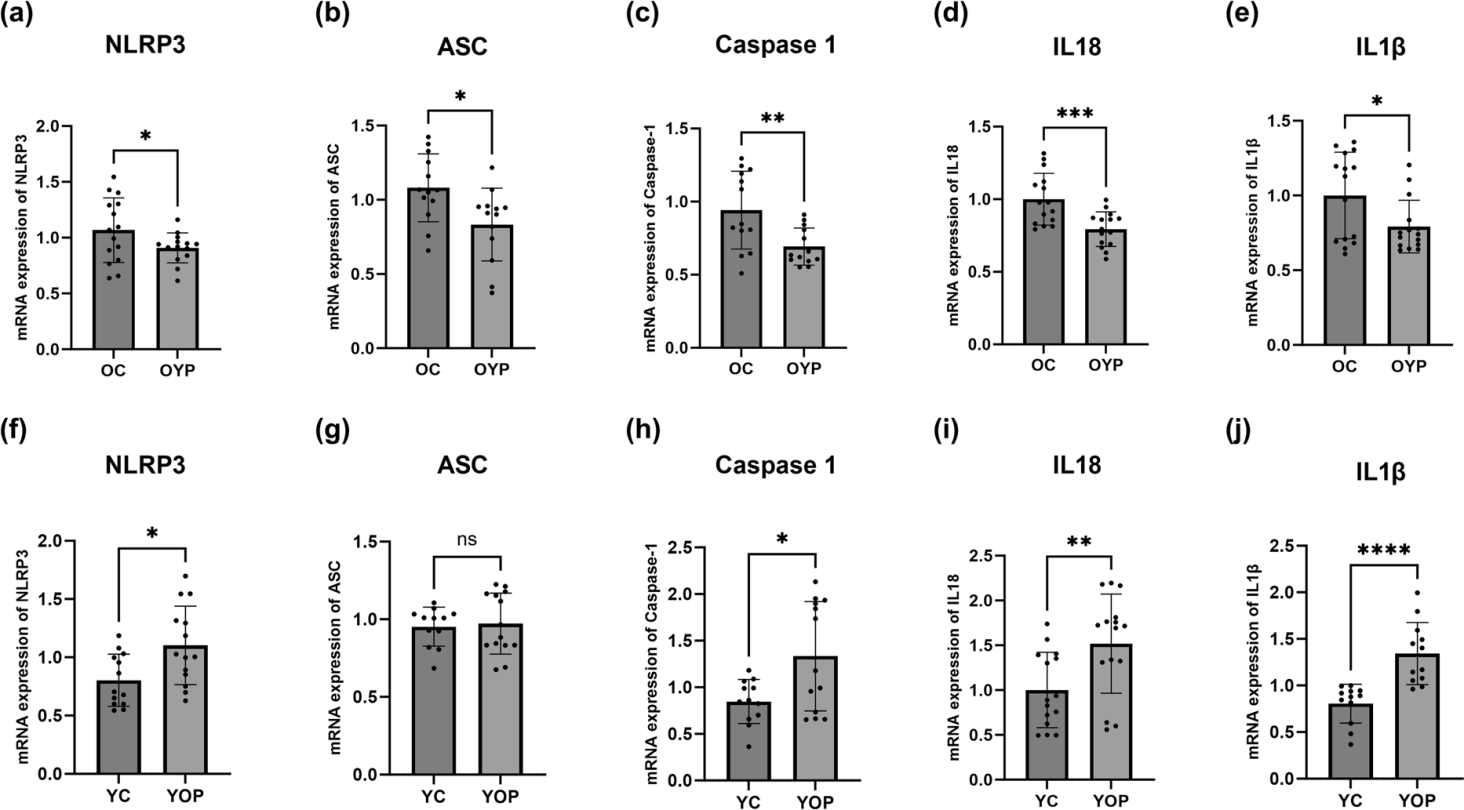
The relative mRNA expression levels in inflammasome-related gene expressions. (**a-f**) mRNA expression levels of NLRP3, (**b-g**) mRNA expression levels of ASC, (**c-h**) mRNA expression levels of Caspase-1, (**d-i**) mRNA expression levels of IL18, and (**e-j**) mRNA expression levels of IL1β. All data are shown as Mean ± SEM (n = 5 and 3 replicates); p values are derived using Tukey’s Multiple Comparisons Test followed by One-way ANOVA. Statistically significant differences are indicated as follows: ns (not significant, p > 0.05); * (significant, p < 0.05); ** (highly significant, p < 0.01); *** (very highly significant, p < 0.001); and **** (extremely significant, p < 0.0001).YC (young control); YOP (young with old plasma); OC (old control); OYP (old with young plasma)

In this study, we also investigated the effects of plasma exchange on the expression of NLRP3 inflammasome genes by comparing mRNA gene expression levels. As shown in Fig. 5a–f, the expression of the NLRP3 gene was significantly upregulated in the YOP group compared to the YC group, indicating an inflammatory response induced by old plasma in young rats. Conversely, the OC group exhibited high levels of NLRP3 expression, consistent with the chronic inflammatory state associated with aging. Notably, the OYP group showed marked reductions in NLRP3 expression compared to the OC group, indicating that young plasma may mitigate inflammaging in old rats. In Fig. 5b–g, the ASC gene expression increased significantly in the YOP group compared to the YC group, suggesting enhanced NLRP3 inflammasome activation due to old plasma. The OC group presented high ASC expression levels, reflecting the chronic inflammatory state in aged rats, whereas the OYP group showed a significant decrease in ASC expression compared to the OC group. Caspase-1 gene expression, depicted in Fig. 5c–h, followed a similar trend with elevated levels in the YOP group compared to the YC group, supporting the pro-inflammatory effects of old plasma on young rats. Elevated caspase-1 levels were observed in the OC group, aligning with the known inflammatory profile of aged rats. The OYP group exhibited a substantial reduction in caspase-1 expression compared to the OC group, indicating that young plasma can attenuate the NLRP3 activation of the inflammasome pathway in old rats. IL-1β, shown in Fig. 5d–i, was significantly upregulated in the YOP group compared to the YC group, suggesting enhanced inflammatory signaling due to old plasma. The OC group had high levels of IL-1β, consistent with the heightened inflammatory state in aged rats, while the OYP group showed a significant reduction in IL-1β expression compared to the OC group. Finally, IL-18 expression is illustrated in Fig. 5e–j. Baseline IL-18 expression was observed in the YC group, but the YOP group exhibited a marked increase in IL-18 expression, indicating an inflammatory response triggered by old plasma. High levels of IL-18 expression were detected in the OC group, characteristic of the inflammatory milieu in aging. However, a significant decrease in IL-18 expression was noted in the OYP group compared to the OC group, highlighting the potential of young plasma to reverse inflammaging.

### Effects of plasma exchange on hematological parameters

**Fig. 6:**
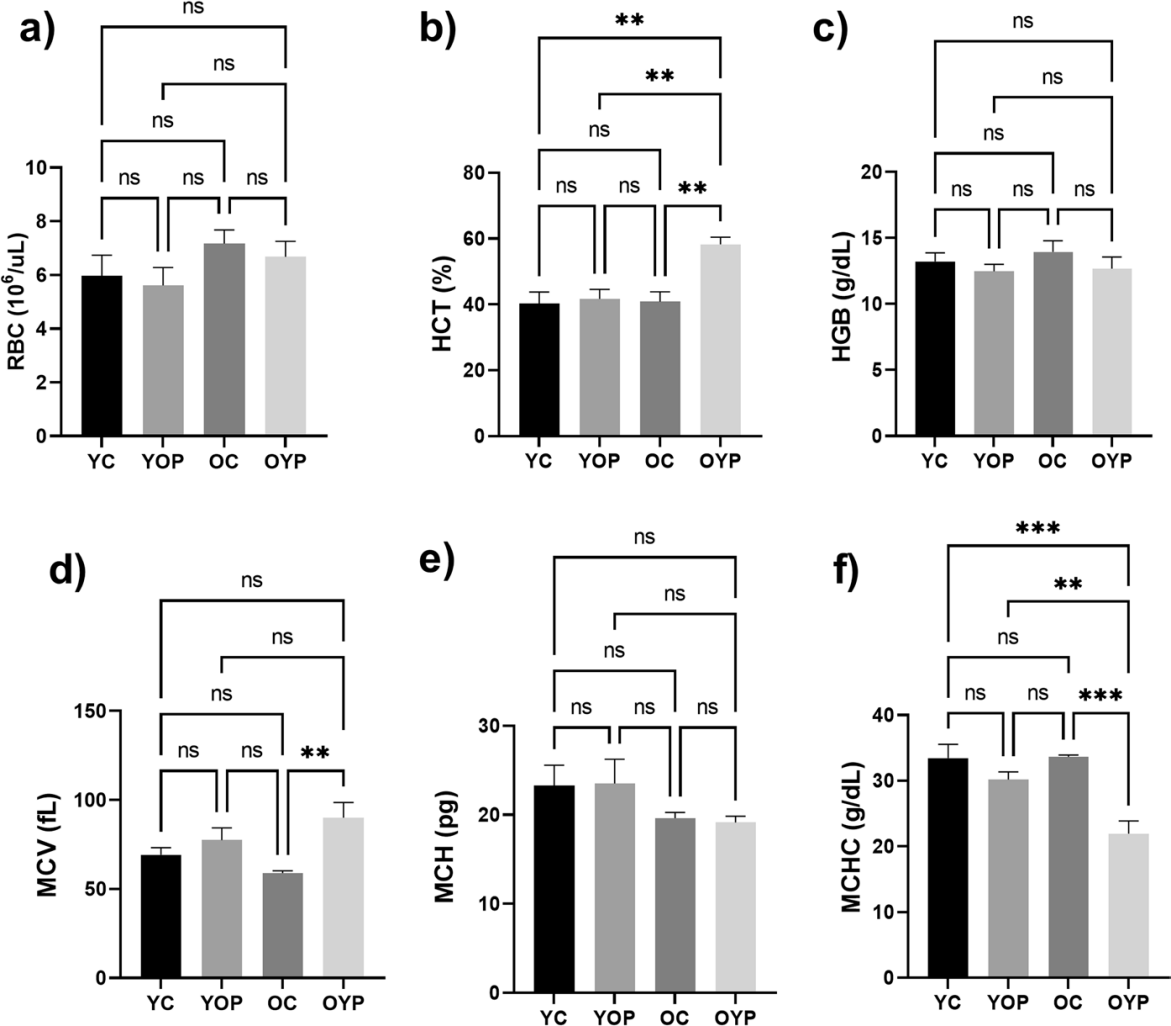
Graphs showing the hematological parameters of (**a**) RBC, (**b**) HCT, (**c**) HGB, (**d**) MCV, (**e**) MCH, and (**f**) MCHC levels in YC, YOP, OC and OYP groups. YC (young control); YOP (young with old plasma); OC (old control); OYP (old with young plasma) were stated as p ≤ 0.05 *, p ≤ 0.01 **, p ≤ 0.001 ***, and p ≤ 0.0001 ****. Data were analyzed using One-way ANOVA and/or unpaired t-test, and the significance levels were stated as p ≤ 0.05 *, p ≤ 0.01 **, p ≤ 0.001 ***, and p ≤ 0.0001 ****. Results are presented as mean ± SEM (standard error of the mean). RBC (Red blood cell, 10.^6^/uL); HCT (hematocrit, %); HGB (hemoglobin, g/dL); MCV (mean corpuscular volume, fL); MCH (mean corpuscular hemoglobin, pg); MCHC (mean corpuscular hemoglobin concentration, g/dL)

Hematological parameters are important data in the preclinical studies of aged-related diseases (Zhang et al. 2018). In this context, our study evaluated the variations of plasma exchange on hematological changes in all groups. The hematological parameters of (a) RBC, (b) HCT, (c) HGB, (d) MCV, (e) MCH, and (f) MCHC levels analyzed for all groups were given in Fig. 6. There were no changes in RBC, HGB, and MCH values between all groups, whereas a significant increase was observed in MCV values in the OYP group compared to the OC group (Fig. 6d, **p ≤ 0.01). MCHC values showed a significant increase in the OC group compared to the OYP group (Fig. 6f, ***p ≤ 0.001), and a significant increase in the YOP group compared to the OYP group (**p ≤ 0.01).

**Fig. 7:**
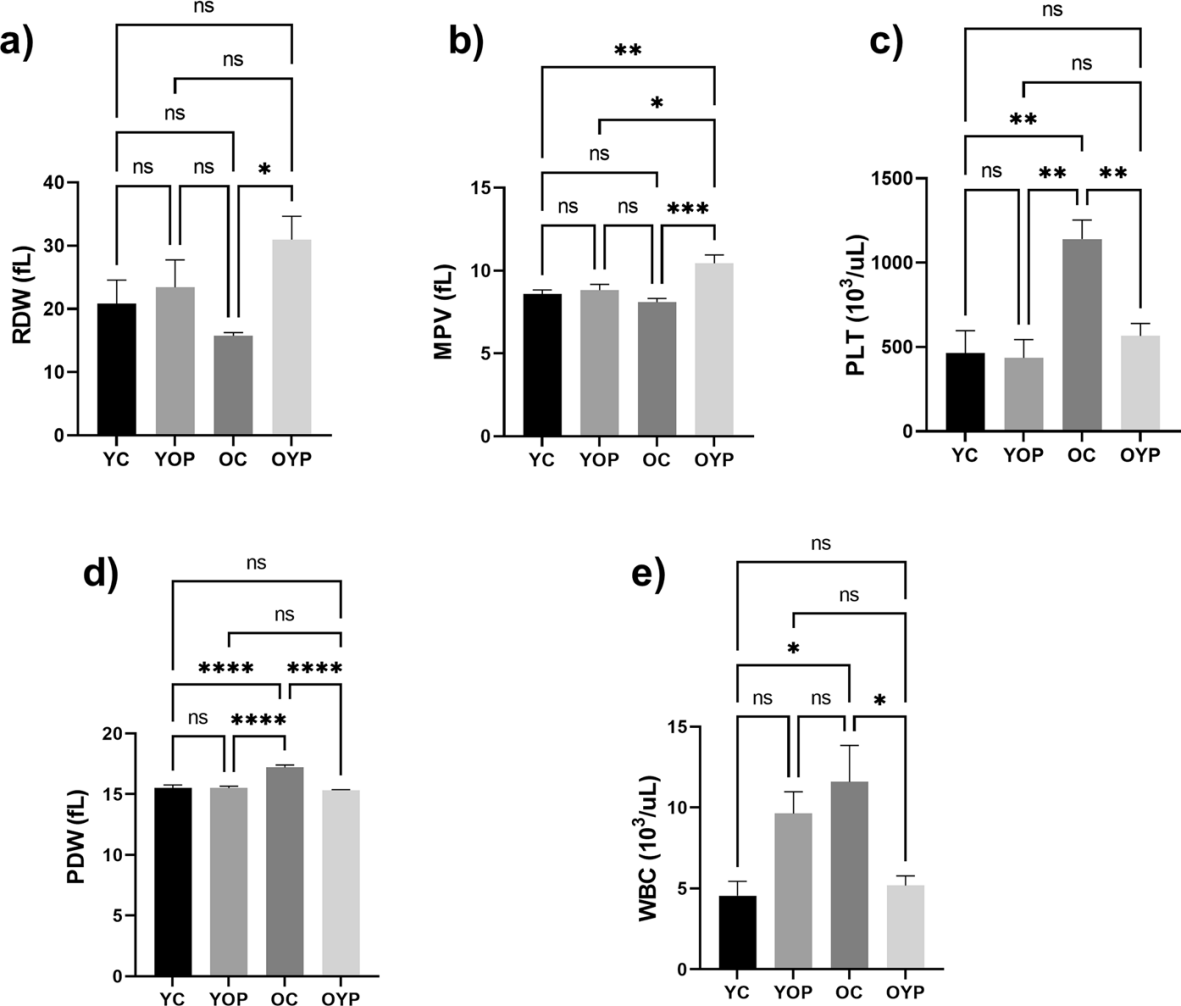
Graphs showing hematological parameters of (**a**) RDW, (**b**) MPV, (**c**) PLT, (**d**) PDW, (**e**) WBC levels in YC, YOP, OC and OYP groups. YC (young control); YOP (young with old plasma); OC (old control); OYP (old with young plasma) were stated as p ≤ 0.05 *, p ≤ 0.01 **, p ≤ 0.001 ***, and p ≤ 0.0001 ****. Data were analyzed using One-way ANOVA and/or unpaired *t*-test, and the significance levels were stated as p ≤ 0.05 *, p ≤ 0.01 **, p ≤ 0.001 ***, and p ≤ 0.0001 ****. Results are presented as mean ± SEM (standard error of the mean). RDW (Red cell distribution width, fL); MPV (Mean platelet volume, fL); PLT (Platelets, 10^3^ /uL); PDW (Platelet distribution width, fL); WBC (White blood cell, 10.^3^ /uL)

In addition, the other hematological parameters of (a) RDW, (b) MPV, (c) PLT, (d) PDW, and (e) WBC values are shown in Fig. 7. A significant increase was observed in RDW values in the OYP group compared to the OC group (Fig. 7a, p ≤ 0.05 *). There was a significant increase in MPV level in the OYP group compared to OC, YC, and YOP groups, respectively (Fig. 7b, p ≤ 0.001 ***, p ≤ 0.01 **, p ≤ 0.05 *). On the other hand, when PLT and PDW levels were analyzed (Fig. 7c and Fig. 7d), significant increases were observed in OC group compared to OYP, YC and YOP groups, respectively (p ≤ 0.01 **, and ****p ≤ 0.0001), and when WBC levels were evaluated (Fig. 7e), it was concluded that OC group showed a significant increase compared to YC and OYP groups (p ≤ 0.05 *).

## Discussion

Previous research has established that young plasma can mitigate systemic inflammation and cellular senescence by modulating key signaling pathways, including NF-κB, AMPK/ULK1, JAK/STAT3, and aged-related cellular responses, across various tissues (A. Liu et al. 2019; Wei et al. 2023; Y. Zhao et al. 2020). However, direct evidence linking young plasma to suppression of inflammasome-mediated inflammation and necroptosis in the aged liver has remained lacking, underscoring the rationale for the present study. Our study extends this knowledge by demonstrating, for the first time, that young plasma not only reduces inflammatory cytokine expression but also attenuates NLRP3 inflammasome activation and necroptotic cell death in aging hepatic tissue. These findings provide mechanistic insight into the multi-faceted anti-inflammatory and cytoprotective effects of young plasma, suggesting its potential as a therapeutic modality for targeting inflammaging and necrotic injury in age-associated liver dysfunction.

Several bioactive molecules present in young plasma have been proposed to mediate its anti-inflammatory and anti-fibrotic effects. Among these, growth differentiation factor 11 (GDF11) has been reported to improve tissue regeneration and reduce fibrosis in aging models by modulating TGF-β signaling (Sinha et al. 2014). Additionally, tissue inhibitor of metalloproteinases 2 (TIMP2) has been identified as a key regulator of extracellular matrix remodeling and has been linked to reduced collagen deposition and attenuation of fibrogenesis (Castellano et al. 2017). Moreover, exosomes derived from young plasma are known to carry a range of microRNAs and proteins that can suppress pro-inflammatory cytokine mediators (such as IL-1β, TNF-α) and inhibit hepatic stellate cell activation, thereby dampening the fibrotic response (B.-R. Lee et al. 2018; Rodrigues et al. 2024). While our study primarily focused on the NLRP3 inflammasome and necroptosis pathways, it is plausible that these and other molecular factors within young plasma collectively contribute to the observed reduction in hepatic inflammation and fibrosis. Age-related liver inflammation is a multifaceted process that causes damage to liver tissue and reduced functionality (Sabira Mohammed et al. 2021a, b). Aged liver cells exacerbate aging-related inflammation by secreting proinflammatory cytokine cascade that further impair liver function and predispose the organ to fibrosis (Dhuriya & Sharma 2018). In a previous study, Hekimoğlu et al. evaluated the connective tissue changes in the livers of young and adult rats using MT staining, and found that young rats exhibited normal histologic connective tissue structures, while adult rats showed significantly increased collagen density due to aging (Hekimoğlu et al. 2024). In another study, Yang et al. investigated the mechanisms underlying liver aging and reported a significant increase in fibrotic areas in older monkeys compared to younger ones (S. Yang et al. 2024). These previous studies suggested that collagen density in liver tissue increases because of aging-related inflammation in the liver. In this context, our data is consistent with previous findings. In addition, when we evaluated the effects of young and old plasma on age-associated collagen density in liver tissue, we found that young plasma could modulate the inflammation pathway and reduce age-associated collagen changes induced by inflammatory responses. In contrast, aged plasma may promote fibrosis in young rats by increasing collagen density in liver tissue because of the aged-related inflammatory molecules it contains. These results suggest that young plasma may prevent age-related liver fibrosis.

Aging is associated with an accumulation of misfolded proteins due to a decline in proteostasis and increased endoplasmic reticulum (ER) stress, which has been identified as a key driver of chronic liver injury and fibrosis (Hetz & Mollereau 2014; Rabek et al. 2003). Persistent ER stress leads to activation of the unfolded protein response (UPR), which, when dysregulated, promotes inflammatory signaling and induces hepatic stellate cell (HSC) activation—a critical step in fibrogenesis. Misfolded protein aggregates can also directly stimulate Kupffer cells and other liver-resident immune cells, exacerbating the inflammatory milieu and further accelerating fibrotic progression (Bárcena et al. 2021; Erickson et al. 2006; Gagliano et al. 2007; Maiers & Malhi 2019; Rabek et al. 2003). Thus, the interplay between protein misfolding, ER stress, and fibrogenic signaling forms a pivotal axis in the pathogenesis of aging-related liver dysfunction. Our findings suggest that interventions such as young plasma therapy may indirectly alleviate fibrotic processes by modulating upstream inflammatory pathways that are linked to protein misfolding stress. Specifically, we observed that young plasma administration significantly attenuated NLRP3 inflammasome activation and reduced necroptosis marker expression in aged hepatic tissue. Given that sustained activation of the NLRP3 inflammasome is known to amplify ER stress and exacerbate the accumulation of misfolded proteins, its suppression by young plasma likely contributes to the restoration of proteostasis. This downregulation of inflammasome-driven inflammation reduces the paracrine activation of hepatic stellate cells (HSCs), which are central mediators of extracellular matrix deposition and fibrosis. Therefore, by dampening both inflammasome-mediated inflammatory cascades and necroptotic cell death, young plasma may help to break the vicious cycle linking protein misfolding, chronic inflammation, and fibrogenesis in the aging liver. These mechanistic insights underscore the potential of young plasma as a multifaceted therapeutic approach to counteract liver inflammaging and fibrosis.

Age-related chronic inflammation implicates activation of the NLRP3 inflammasome complex in liver aging (Wree et al. 2014). Furthermore, studies have emphasized the role of caspase 1, IL-1β, and IL-18 in NLRP3 inflammasome pathway activation in aging-related liver injury (S. Mohammed et al. 2021a, b; Selvarani et al. 2023). Researchers found high levels of NLRP3 inflammasome pathway components expressed in age-related liver diseases (Baba et al. 2024; Ma et al. 2018; Wree et al. 2014). Our study showed that young plasma can suppress the inflammasome axis by significantly reducing the critical increases in NLRP3, ASC, caspase-1, and IL-1β expression levels in aged liver tissue because of aging-related inflammation. In addition, qPCR results obtained at the gene level were correlated with our IHC data. Hekimoglu et al. found significant increases in TNF-α and NF-κB levels in the liver of 6-week-old and 10-month-old rats (Hekimoğlu et al. 2024). In addition, Yang et al. also reported significant increases in the expression of inflammation-related cytokines (TNF-, IL-1β, and IL-6) and upregulation of aging-related genes in the liver of aged monkeys (S. Yang et al. 2024). Consistent with previous studies, our findings support the increased NLRP3 inflammasome activation in aged rat liver tissue because of inflammaging. Our study also provides important insights that young plasma attenuates aged liver injury by preventing activation of the NLRP3 inflammasome complex and associated genes.

Aged liver cells promote the activation of RIPK1, RIPK3, and MLKL, producing inflammatory stimulators in the necroptosis (Hao et al. 2023; Tran et al. 2024). Aging may exacerbate the inflammatory state since this induces both necroptosis and the NLRP3 inflammasome complex. Mohammed et al. observed that aged mice showed significantly higher levels of necroptosis-related molecules in their livers than young mice, with this increase becoming more significant after 18 months (S. Mohammed et al. 2021a, b). Stahl et al. reported a close age-related correlation between aging and liver inflammation, finding that some age-related pathologies were significantly more prevalent in 19-month-old mice than in 3-month-old mice (Stahl et al. 2020). On the other hand, in another study, a Western-blotting analysis of RIP3 protein, a critical regulator of necroptosis, also reported that necroptosis expression levels in adult rats were lower than in young rats (Hekimoğlu et al. 2024). In the present study, we observed that young plasma significantly decreased necroptosis signaling, and inhibited the liver inflammation. In addition, aged plasma may have triggered inflammation and fibrosis in liver tissue by activating necroptosis signaling in young rats. Our findings suggest that aging-associated inflammation may cause liver injury by activating the necroptosis pathway and NLRP3 inflammasome. As can be seen, our findings are parallel to those of the reported studies. The conflicting data on necroptosis signaling and age-related organ damage suggest that the experimental conditions or animal species used in published studies lead to heterogeneity in expression levels. This study demonstrated that the interplay of the necroptosis pathway and the NLRP3 inflammasome complex can accelerate age-related liver injury, and young plasma may suppress both the necroptosis and NLRP3 inflammasome pathways.

Interactions between TNF-α and VEGFR2 are involved in the control of necroptosis signaling depending on the severity of inflammation in aged liver (Shibuya 2015). TNF-α is involved in initiating necroptosis in liver cells due to aging. Previous studies indicate a TNF-α-mediated link between necroptosis and hepatic fibrosis during aging. This is because TNF-α may activate VEGFR2, which is elevated in aging and has been reported to lead to chronic inflammation and fibrosis in the aged liver (Coulon et al. 2012; Grunewald et al. 2021). Changes in TNF-α and VEGFR2 expressions in the aged liver may be elevated in inflammation-induced liver injury. Furthermore, young plasma might mediate TNF-α-mediated necroptosis signaling by decreasing these increases. On the other hand, aged plasma seems to increase inflammaging by increasing TNF-α and VEGFR2 levels in young rat liver. All these findings suggest that young plasma can attenuate age-related liver injury by modulating TNF-α and VEGFR2 expression levels.

Protein secondary structures, such as α-helices and β-hairs, are essential elements of protein architecture that influence a protein’s stability, function, and interaction with other molecules (Stollar & Smith 2020). The underlying causes of many chronic diseases, such as aging, are increasingly linked to disruptions in the mechanisms that regulate the structure of these proteins. (Diaz-Villanueva et al. 2015). Inflammation is a complex biological response to injury or the aging process, regulated by signaling molecules which are usually related to inflammatory proteins (H. Zhao et al. 2021). These cytokines’ correct folding and structural integrity, determined by their secondary structure, are crucial for their function (Cui & Lisi 2021). The native structure of a folded protein is shaped by a delicate balance of interactions at various levels. Short-range local interactions dictate the intrinsic conformational tendencies of individual amino acids, while medium-range interactions stabilize specific regions of secondary structure, such as α-helices and β-sheets. Nonlocal tertiary interactions ultimately define the overall three-dimensional fold of the protein (Smith et al. 1996). However, a random coil is a polymer chain that coils randomly in three dimensions following the Gaussian probability function. In protein chemistry, however, the term random coil has a more specific meaning: It describes a situation where the conformation of each amino acid residue in the protein’s backbone, defined by its phi and psi angles, is independent of the conformations of neighboring residues (Baldwin & Zimm 2000).

Aging involves the disruption of fundamental cellular processes. Aging cells experience a decline in protein homeostasis (proteostasis) mechanisms, resulting in the accumulation of damaged and misfolded proteins (Taylor & Dillin 2011). Lacking a stable tertiary structure, intrinsically disordered proteins (IDPs) and intrinsically disordered regions (IDRs) serve as adaptive hubs in signaling pathways and have the potential to regulate a variety of processes, including those linked to aging (Hipp et al. 2019; Manyilov et al. 2023). As aging progresses, the quality control of proteostasis decreases, leading to the accumulation of damaged proteins. Under these stressful conditions, chaperones that inherently contain IDRs lose their specificity, leading to an increase in cellular ‘noise’ or loss of weak interactions. As a result, the proper protein–protein interaction (PPI) network is disrupted, leading to an overall impairment in cellular and organismal function (Lindner & Demarez 2009).

Necroptosis involves a multiprotein complex known as the necrosome where RIPK1 and RIPK3 activity is required. The secondary structures of RIPK1 and RIPK3, particularly in their kinase domains, are critical for their interaction and activation (Berghe et al. 2014). Misfolding or alterations in these domains can disrupt necrosome structure, impairing necroptosis. Furthermore, proteins such as MLKL, which form pores in the plasma membrane to carry out necroptosis, rely on specific secondary structures to oligomerize and translocate to the membrane (Martinez-Osorio et al. 2023). According to our results, the blood plasma exchange between old and young rats resulted in severe conformational changes in protein secondary structures. Aged rats receiving young plasma increased in several β-sheet elements and α-helices like the young rat control group. However, young rats receiving aged plasma showed highly disordered structures (random helices), β-turns, 3_10_ helices, and loops connecting helices, similar to the aged rat control group. Young plasma effectively reverses these conformational changes in the proteins of old rats, whereas old plasma causes a worsening effect on protein secondary structures in young rats. The observed increase in disordered structures in young rats receiving aged plasma is of particular concern, as a similar increase in random helices has been associated in previous studies with pathological conditions in rats, such as epileptic seizures and systemic inflammation (Cakmak-Arslan et al. 2024; Turker et al. 2014). The spectroscopic results from our study underscore the potential risk of aging-associated conformational changes of proteins contributing to the progression of inflammation-related liver injury, possibly through mechanisms involving destabilization of protein structures and formation of random helices.

Hematologic analyses are crucial for diagnosing age-related organ dysfunctions and evaluating the body’s response to treatment in age-related studies (Delwatta et al. 2018). Due to various factors, such as dehydration and hematologic markers, HBG and RBC levels may remain unchanged, while HCT levels may increase, leading to a decrease in plasma volume and an increase in the concentration of red blood cells. Even if the total number of erythrocytes and hemoglobin levels remain the same, HCT can increase due to changes in the size or shape of erythrocytes. In some cases, HCT has been reported to be slightly elevated within normal ranges without any indication of an underlying medical condition (Jacob Filho et al. 2018). In our study, the significant increase in HCT in young rats receiving aged plasma compared to the other groups may be due to related conditions. On the other hand, age-related changes in the structure and function of blood vessels may also cause inflammation and increase platelet production (Medvedev 2017). Therefore, we observed that the increases in platelet levels in aged rats were reduced by young plasma treatment. The results suggest that young plasma may modulate platelet synthesis. Furthermore, in the impairment of the immune system, which is characterized by changes in immune system function with aging, the activity of immune cells may decrease, and their propensity to take part in inflammation may increase. This has been reported to lead to an increase in leukocyte levels as part of chronic inflammation (Kojima et al. 1999). In addition, cellular damage molecules in the aging process are known to induce leukocyte production by triggering immune system responses (Hussain 2014). In the present study, young plasma reduced the high white blood cell levels in the aged rats. Our data underline that anti-inflammatory and immunomodulatory molecules present in young plasma may play an important role in managing the inflammation that accompanies aging.

Our findings demonstrate that young plasma administration suppressed both NLRP3 inflammasome activation and necroptosis marker expression in aged livers, indicating that this therapy concurrently modulates interconnected inflammatory and cell death pathways. This dual impact underscores the multifaceted therapeutic potential of young plasma in disrupting the pathological processes driving hepatic inflammaging and fibrosis. However, several important limitations must be acknowledged to provide a balanced interpretation of these promising results. Although our data reveal significant modulation of inflammatory and necroptotic responses, the mechanistic insights are primarily derived from protein secondary structures, immunohistochemical, and gene expression analyses; thus, deeper molecular investigations are required to fully elucidate the underlying pathways. Moreover, the study was conducted exclusively in an animal model, which may not fully recapitulate the complexity of age-related liver inflammation in humans. Safety considerations also present critical challenges: The ethical implications of sourcing plasma from young donors, even within controlled experimental settings, raise concerns regarding donor welfare and translatability to human therapy. Additionally, the safety profile of heterochronic plasma transfusion—including the risks of immune reactions, pathogen transmission, and long-term adverse effects—remains insufficiently characterized and warrants comprehensive evaluation prior to clinical application (Biller-Andorno 2016; Kheifets & Braithwaite 2019; M.-N. Liu et al. 2024). Future research should prioritize the identification and isolation of key bioactive components and their signaling pathways to facilitate the development of targeted, safe, and ethically sustainable interventions for age-related inflammatory conditions.

## Conclusion

Our results suggest that increased activation of the NLRP3 inflammasome and necroptosis may contribute to age-related liver injury. The conformational changes in the secondary structures of the proteins obtained from our study were confirmed by molecular and immunohistochemical analysis. The data mechanistically elucidate that young plasma transfer may attenuate age-related liver damage by focusing on the necroptosis and NLRP3 inflammasome axis. Furthermore, this study shed light on the strategic potential of young plasma in treating other age-related diseases.

## Supplementary Information

Below is the link to the electronic supplementary material.

Supplementary file1 (DOCX 484 KB)

## Data Availability

All data generated and/or analyzed during the current study are available from the corresponding author on reasonable request.

## Code availability

There is not any custom computer code or algorithm used to generate the results reported in the manuscript.
