# Supplementary material for: Therapeutic potential of young plasma in reversing age-related liver inflammation via modulation of NLRP3 inflammasome and necroptosis
Source: Biogerontology. 2025 May 26;26(3):117. doi: 10.1007/s10522-025-10260-9 (PMC12106525; doi:10.1007/s10522-025-10260-9)
Supplement: Supplementary file 1 — Supplementary file1 (DOCX 484 KB) [file 10522_2025_10260_MOESM1_ESM.docx]

**Supplementary Information**


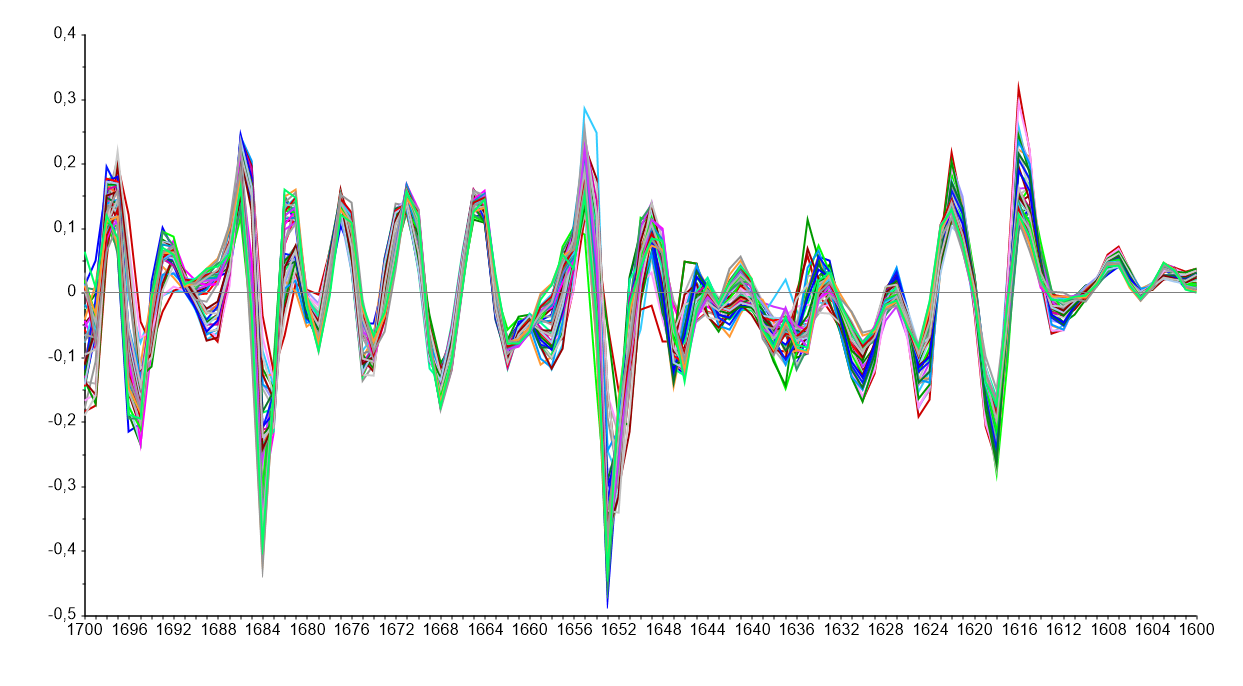


**Figure S1**. The line plot of the pre-treated dataset used in Principal Component Analysis which was conducted on liver infrared (IR) spectra after different pretreatment steps using The Unscrambler® X 10.3 (CAMO Software AS, Norway) software. The raw IR data were subjected to baseline offset transformation in the 4000–650 cm^−1^ spectral window. The second data derivatives were obtained using the Savitzky-Golay transformation method in the 1700–1600 cm^−1^ spectral window of Amide I protein. The second derivative spectra were transformed using the unit vector normalization method.

**Table S1.** The main X-loadings of the PC-1 component demonstrating main protein secondary structures in the 1700–1600 cm^−1^ spectral window of Amide I protein.

| **Main X-loadings** | **PC-1** | **Protein Secondary Structures** |
| --- | --- | --- |
| 1696 | - 0,25 | Antiparallel β-Sheet (Barth & Zscherp 2002; Haris & Severcan 1999) |
| 1684 | - 0,36 | Intermolecular β-Sheet (Severcan & Haris 2003) |
| 1682 | 0,21 | β-Turn (Usoltsev et al. 2019) |
| 1675 | 0,12 | β-Turn (Kong & Yu 2007) |
| 1670 | 0,09 | β-Turn (Severcan & Haris 2003) |
| 1663 | 0,07 | 3_10_ Helix (José Luis R Arrondo & Goñi 1999; D. Silva et al. 2002; Kong & Yu 2007) |
| 1657 | 0,15 | 3_10_ Helix (José Luis R Arrondo & Goñi 1999; D. Silva et al. 2002) |
| 1653 | - 0,33 | α-Helix (Jose Luis R Arrondo et al. 1993; Severcan & Haris 2003) |
| 1648 | 0,10 | Random Coil (unordered elements) (Cakmak‐Arslan et al. 2024; Kong & Yu 2007; Usoltsev et al. 2019) |
| 1646 | - 0,11 | α-Helix (Haris & Severcan 1999) |
| 1642 | 0,07 | Random Coil (unordered elements) (Goormaghtigh et al. 1990; Haris & Severcan 1999) |
| 1637 | 0,04 | Loop structures connecting helices (Severcan & Haris 2003) |
| 1635 | - 0,12 | β-Sheet (Cobb et al. 2020) |
| 1627 | - 0,05 | β-Sheet (Kong & Yu 2007) |
| 1624 | 0,13 | β-Sheet (Cobb et al. 2020; Gurbanov et al. 2016) |
| 1616 | - 0,17 | Intermolecular β-Sheet (Severcan & Haris 2003; Usoltsev et al. 2019) |

**References**

Arrondo JLR, Goñi FM (1999) Structure and dynamics of membrane proteins as studied by infrared spectroscopy. Progress in biophysics and molecular biology 72: 367–405.

Arrondo JLR, Muga A, Castresana J, Goñi FM (1993) Quantitative studies of the structure of proteins in solution by Fourier-transform infrared spectroscopy. Progress in biophysics and molecular biology 59: 23–56.

Barth A, Zscherp C (2002) What vibrations tell about proteins. Q. Rev. Biophys. 35: 369–430.

Cakmak‐Arslan G, Kaya Y, Mamuk S, Akarsu ES, Severcan F (2024) The investigation of the molecular changes during lipopolysaccharide‐induced systemic inflammation on rat hippocampus by using FTIR spectroscopy. Journal of Biophotonics 17: e202300541.

Cobb JS, Zai-Rose V, Correia JJ, Janorkar AV (2020) FT-IR spectroscopic analysis of the secondary structures present during the desiccation induced aggregation of elastin-like polypeptide on silica. ACS omega 5: 8403–8413.

D. Silva RG, Yasui SC, Kubelka J, Formaggio F, Crisma M, Toniolo C, Keiderling TA (2002) Discriminating 310‐from α‐helices: Vibrational and electronic CD and IR absorption study of related Aib‐containing oligopeptides. Biopolymers: Original Research on Biomolecules 65: 229–243.

Goormaghtigh E, Cabiaux V, RUYSSCHAERT JM (1990) Secondary structure and dosage of soluble and membrane proteins by attenuated total reflection Fourier‐transform infrared spectroscopy on hydrated films. Eur. J. Biochem. 193: 409–420.

Gurbanov R, Bilgin M, Severcan F (2016) Restoring effect of selenium on the molecular content, structure and fluidity of diabetic rat kidney brush border cell membrane. Biochimica et Biophysica Acta (BBA)-Biomembranes 1858: 845–854.

Haris PI, Severcan F (1999) FTIR spectroscopic characterization of protein structure in aqueous and non-aqueous media. J Mol Catal B-Enzym 7: 207–221. <https://doi.org/10.1016/S1381-1177(99)00030-2>

Kong J, Yu S (2007) Fourier transform infrared spectroscopic analysis of protein secondary structures. Acta biochimica et biophysica Sinica 39: 549–559.

Severcan F, Haris PI (2003) Fourier transform infrared spectroscopy suggests unfolding of loop structures precedes complete unfolding of pig citrate synthase. Biopolymers: Original Research on Biomolecules 69: 440–447.

Usoltsev D, Sitnikova V, Kajava A, Uspenskaya M (2019) Systematic FTIR spectroscopy study of the secondary structure changes in human serum albumin under various denaturation conditions. Biomolecules 9: 359.
